# Impact of proactive risk-prevention nursing interventions on the recovery of ICU patients with ventilator-associated pneumonia

**DOI:** 10.3389/fmed.2025.1706452

**Published:** 2026-01-05

**Authors:** Zhangyan Wei, Xiaomei Liu

**Affiliations:** 1 Department of ICU, Linquan County People’s Hospital, Fuyang, Anhui, China; 2 Department of Head and Neck Surgery, Ganzhou Cancer Hospital, Ganzhou, Jiangxi, China

**Keywords:** inflammatory cytokines, intensive care unit, mechanical ventilation, proactive risk-prevention nursing, psychological comfort, ventilator-associated pneumonia

## Abstract

**Background and objective:**

Ventilator-associated pneumonia (VAP) is a common complication in patients undergoing mechanical ventilation. For patients with VAP, in addition to active treatment, targeted nursing interventions are required. However, routine nursing care often overlooks various risk factors that may arise during the patient’s recovery process. Consequently, this study aims to reveal the impact of proactive risk-prevention nursing interventions on the recovery of intensive care unit (ICU) patients with VAP.

**Methods:**

This study was a randomized controlled trial conducted in accordance with the CONSORT guidelines. Using purposive sampling, 90 ICU patients with VAP were randomly classified into a control group and an observation group, with 45 patients in each. The control group received routine nursing interventions, while the observation group was treated with proactive risk-prevention nursing interventions. Primary outcome measures: The mechanical ventilation time, ICU stay, and body temperature recovery time were compared. Secondary outcome measures: White blood cell count (WBC), neutrophil count (NC), inflammatory cytokine levels, psychological comfort levels (assessed using the General Comfort Questionnaire), and patient satisfaction with nursing interventions were compared.

**Results:**

The observation group exhibited shorter mechanical ventilation duration, ICU stay, and body temperature recovery compared to the control group (*P* < 0.05). Additionally, the observation group demonstrated greater improvement in WBC, NC, CRP, IL-4, PCT, and GCQ scores across all dimensions, along with higher patient satisfaction, compared to the control group (all *P* < 0.05).

**Conclusion:**

Proactive risk-prevention nursing interventions can reduce mechanical ventilation duration and ICU stay, decrease inflammatory cytokine levels, improve psychological comfort, and enhance patient satisfaction with nursing interventions in ICU patients with VAP.

## Introduction

Ventilator-associated pneumonia (VAP) refers to an infection of the lung tissue that occurs in individuals undergoing invasive mechanical ventilation for a minimum of 48 h, representing a form of hospital-acquired pneumonia within intensive care unit (ICU). It remains one of the most prevalent infections affecting patients who depend on invasive mechanical ventilation (1). Several factors contribute to the development of VAP. These factors include the use of endotracheal tubes, the buildup of secretions around the tube cuff, inadequate oral hydration with dry mouth conditions, reduced cough reflex efficiency, difficulties in clearing pharyngeal and oral secretions, and insufficient oral hygiene practices (2). Among these factors, the use of an endotracheal tube disrupts the natural protective reflexes of the upper airway, impairs effective coughing, irritates the respiratory mucosa, elevates mucus production, and facilitates the microaspiration of contaminated secretions from the oropharynx (3). While traditional nursing methods remain prevalent in VAP treatment, their clinical efficacy and operational productivity prove inadequate, underscoring the imperative to explore superior care alternatives (4).

Given that VAP prolongs ICU stays, extends mechanical ventilation time, raises treatment expenses, and elevates mortality rates, developing effective preventive strategies against VAP remains a clinical priority (5, 6). A 2022 retrospective study indicates that effective healthcare team collaboration supports the implementation of active prevention measures, enables prompt identification of VAP, and facilitates timely intensive care (7). In this context, the proactive risk-prevention nursing model, which embodies “foresight, systematization, and integration,” has gradually gained attention (8). This approach helps clinicians identify potential process failures, their causes, and consequences, and guides targeted preventive strategies (9). This nursing model emphasizes comprehensive assessment, proactive intervention, and continuous optimization of nursing plans before patient symptoms manifest, aiming to minimize the risk of complications and promote patient recovery (8). A 2021 model validation study showed that proactive risk mitigation helps prevent disease progression while promoting organizational improvements, thereby diminishing both mortality figures and inpatient care expenses (10); meanwhile, a review article published by Meitner et al. stated that proactive nursing strategies facilitates enhanced monitoring of mechanically ventilated patients, enabling timely identification and management of physiological alterations associated with hospital care (11).

Although numerous studies have focused on the prevention and treatment of VAP in ICU patients (12–14), there remains a significant gap in proactive risk-prevention nursing interventions. Existing research still lacks a systematic, comprehensive, and highly targeted proactive risk-prevention nursing intervention system. We aim to fill this gap by constructing and validating the impact of a proactive risk-prevention nursing intervention plan on the recovery of ICU patients with VAP, with the goal of providing a higher-quality and more efficient nursing model for ICU patients with VAP.

## Materials and methods

### Ethics statement

The research was approved by the Ethics Committee of Linquan County People’s Hospital. The participants involved in the study signed the written informed consent forms.

### Study design

This study was a single-center, randomized controlled trial conducted in accordance with the CONSORT guidelines.

### Subjects

Using purposive sampling, 90 patients diagnosed with VAP who received mechanical ventilation in the ICU from October 2022 to October 2023 in the Linquan County People’s Hospital were selected. They were randomly classified into a control group and an observation group, with 45 patients in each.

Inclusion criteria: patients receiving mechanical ventilation in the ICU, meeting the VAP diagnostic criteria (15), and who confirmed to have VAP through clinical symptom observation (fever, cough, increased purulent airway secretions), lung X-ray or CT examination (pulmonary infiltrates, consolidation, or cavitation), laboratory tests showing elevated inflammatory markers such as white blood cells, neutrophils, C-reactive protein (CRP), procalcitonin (PCT), and pathogen culture of sputum and bronchoalveolar lavage fluid (pathogen concentration ≥ 10^5^ CFU/mL); patients with mechanical ventilation duration exceeding 2 days; patients in clinically stable condition; patients newly admitted to the ICU; patients with complete clinical data; patients providing informed consent.

Exclusion criteria: patients with severe pulmonary diseases such as lung cancer or tuberculosis; patients with functional failure of vital organs such as the heart, liver, or kidneys; patients with mechanical ventilation duration less than 48 h; patients with cognitive dysfunction.

### Sample size calculation

Sample size estimation was performed using G*Power 3.1.9.7. Based on the expectation that the nursing intervention would produce significant improvements in primary outcomes such as mechanical ventilation duration and ICU stay, an effect size *d* = 0.8 was selected. With α = 0.05 (two-tailed) and power (1−β) = 0.90, the calculated total sample size was 68 (34 per group). Accounting for a 20% dropout rate, the planned sample size was 82. The study ultimately enrolled 90 patients (45 per group), meeting statistical requirements. No dropouts or withdrawals occurred during the study.

### Randomization process and blinding

Participants were randomly assigned to the control group and the observation group using a randomization method. The specific steps were as follows: a random number table was used to select any number for positioning, and 90 numbers were listed in sequence according to the table. The selected numbers were divided by 2, and the remainders (0 and 1) were used to assign patients to the control and observation groups, respectively. During the randomization process, allocation concealment was implemented by placing the randomization scheme in sequentially numbered, sealed, opaque envelopes (with the envelope number matching the card number inside). Patients were assigned to groups according to their admission order by opening the corresponding envelopes and strictly following the randomization allocation scheme for intervention.

Since this study was a nursing intervention study, blinding of patients and researchers was not feasible. Therefore, blinding was implemented for the outcome collectors and data analysts, who were unaware of the patients’ group assignments. Blinding was lifted after outcome collection and data analysis to ensure the authenticity and reliability of the collected data.

### Methods

Both groups were given invasive mechanical ventilation treatment using SV600 ventilators produced by Shenzhen Mindray Bio-Medical Electronics Co., Ltd., Gunangdong, China. The ventilator settings were as follows: ventilation frequency of 8–14 breaths/min, tidal volume of 6–8 mL/kg, positive end-expiratory pressure of 5–10 cm H_2_O, expiratory time of 1.0–1.2 s, oxygen concentration of 60%, with continuous ventilation for more than 12 h per day.

The control group received routine nursing interventions, including close monitoring of vital signs such as heart rate, blood pressure, and respiratory rate, timely detection and management of potential risks, maintaining a clean environment with good air circulation, adjusting the temperature and humidity to create a conducive rehabilitation environment for patients.

The observation group received proactive risk-prevention nursing interventions in addition to the routine nursing interventions provided to the control group, with specific measures as follows:

(1)    A risk control team was established. The team comprised 3 nurses with more than 5 years of work experience, 1 attending physician, 1 senior nutritionist, and 1 psychologist. All members possessed profound professional knowledge and rich clinical experience. Team members underwent comprehensive training before the intervention, covering clinical manifestations, symptoms, antibiotic use, respiratory support equipment management, and mechanical ventilation techniques related to VAP.
(2)    Nurses followed standardized procedures to identify and quantitatively assess patients’ risk factors, classifying them based on risk assessment results. Risk assessment was conducted using domestic and international professional literature, clinical guidelines, research reports, and case records, combined with patients’ physical condition, medical history, family history, and other potential influencing factors, to develop personalized nursing plans. Risk assessment criteria were classified into three levels: low risk (1–11 points), medium risk (12–19 points), and high risk (20–27 points).
(3)    Corresponding nursing interventions were implemented for patients at different risk levels. For low-risk patients, the importance of respiratory training was emphasized, with gradual training to improve respiratory efficiency. Patients were instructed to maintain a comfortable semi-Fowler’s position and master the technique of whistling breathing to control their breathing rhythm and avoid issues such as hyperventilation or shortness of breath. For medium-risk patients, health education and psychological nursing were strengthened. VAP knowledge was regularly disseminated using videos and images. Emotional needs were addressed through active support and guidance on using music and communication to alleviate distress. Suctioning methods were adjusted flexibly based on individual patient conditions. For high-risk patients, nutritional support therapy was provided during the recovery process, such as nasogastric feeding with nutrients gradually transitioning from low to high concentrations. The nutrient solution was ensured to be comprehensive in composition, heated to an appropriate temperature, and injected through a nasogastric tube. Patients were maintained in a semi-reclining position after nasogastric feeding to facilitate food emptying and were regularly flushed the nasogastric tube to prevent clogging.

### Observation indicators

Primary outcome measures: mechanical ventilation time, ICU stay, and body temperature recovery time were compared.

Secondary outcome measures: (1) White blood cell (WBC) and neutrophil count (NC) before and after nursing interventions: blood samples were collected from patients before and after nursing interventions, and serum or plasma was obtained through centrifugation for the detection of WBC and NC using specific ELISA kits or automated biochemical analyzers.

(2)    Inflammatory cytokine levels before and after nursing interventions: fasting elbow venous blood (3 mL) was drawn from patients before and after nursing interventions, centrifuged at 3000 r/min for 10 min with a centrifugation radius of 10 cm, and serum was obtained. Serum levels of CRP, interleukin-4 (IL-4), and PCT were detected using specific ELISA kits.
(3)    Psychological comfort levels before and after intervention were assessed using the General Comfort Questionnaire (GCQ) in both groups (16): the GCQ was a self-assessment scale comprising four dimensions, including physiological, psychological, sociocultural, and environmental, with a total of 30 items. A 5-point Likert scale, where 1 represented “strongly disagree” and 5 meant “strongly agree.” The physiological dimension had 10 items, with a total score range of 10–50 points. The psychological dimension consisted of 10 items, scoring 10–50 points. The sociocultural dimension comprised 5 items, scoring 5–25 points. The environmental dimension encompassed 5 items, scoring 5–25 points. The total score of the scale ranged from 30 to 150 points, with higher scores indicating higher levels of psychological comfort.
(4)    Patient satisfaction with nursing interventions using a satisfaction questionnaire developed by our hospital: It covered nursing content, nursing attitude, nursing skills, and nursing effects. Based on the scores, satisfaction was categorized into three levels: very satisfied (score > 80), basically satisfied (score 50–80), and unsatisfied (score < 50). Total satisfaction rate was calculated as (very satisfied + basically satisfied)/total number of patients × 100%. The Cronbach’s α for this questionnaire was 0.795.

### Statistical methods

Statistical analysis was processed using SPSS 26.0 software. Before analysis, the Shapiro-Wilk test and Levene test were used to assess the normality and homogeneity of variance of measurement data. Measurement data that were normally distributed and had homogeneous variance were expressed as ($\bar{x}$ ± s). Paired *t*-tests were utilized for comparisons before and after nursing interventions, and independent sample *t*-tests were applied for comparisons between the two groups. Categorical data were presented as Number (*n*)/percentage (%) and processed using chi-square tests. Differences were considered statistically significant at *P* < 0.05.

## Results

### General information

The control group consisted of 25 males and 20 females with a mean age of 48.80 ± 8.79 years (range: 30–59). Among them, 29 received tracheostomy and 16 received tracheal intubation. The observation group included 27 males and 18 females with a mean age of 48.33 ± 8.95 years (range: 31–59), with 28 patients undergoing tracheostomy and 17 receiving tracheal intubation. No statistically significant differences were observed in general information between the two groups (*P* > 0.05), confirming their comparability (Table 1).

**TABLE 1:** Comparison of general information between two groups.

Group	Number of cases	Gender [*n* (%)]		Age (years)	Mechanical ventilation method [*n* (%)]		Body mass index (kg/m^2^)	Smoking history [*n* (%)]	
		Male	Female		Tracheotomy	Tracheal intubation		Yes	No
Control group	45	25 (55.56)	20 (44.44)	48.80 ± 8.79	29 (64.44)	16 (35.56)	22.35 ± 2.07	20 (44.44)	25 (55.56)
Observation group	45	27 (60.00)	18 (40.00)	48.33 ± 8.95	28 (62.22)	17 (37.78)	22.50 ± 2.16	23 (51.11)	22 (48.89)
t/χ^2^		0.182		0.25	0.048		0.336	0.401	
*P*		0.67		0.804	0.827		0.737	0.527	

### Mechanical ventilation time, ICU stay, and body temperature recovery time

The observation group had lower mechanical ventilation time, ICU stay, and body temperature recovery time than the control group (*P* < 0.05) (Table 2).

**TABLE 2:** Mechanical ventilation time, ICU stay, and body temperature recovery time between the two groups ($\bar{x}$ ± s, days).

Group	Number of cases	Mechanical ventilation time	ICU stay	Body temperature recovery time
Control group	45	9.78 ± 1.58	14.67 ± 1.30	6.56 ± 0.84
Observation group	45	6.36 ± 0.80	8.96 ± 0.80	5.29 ± 0.51
t		12.959	25.173	8.66
*P*		<0.001	<0.001	<0.001

### WBC and NC

After nursing interventions, the observation group had lower levels of WBC and NC compared to the control group (*P* < 0.05) (Table 3).

**TABLE 3:** Comparison of WBC and NC between the two groups ($\bar{x}$ ± s).

Group	Number of cases	WBC (10^9^/L)		NC (%)	
		Pre-nursing	Post-nursing	Pre-nursing	Post-nursing
Control group	45	22.91 ± 3.29	12.44 ± 4.16*	85.21 ± 5.75	69.34 ± 5.59*
Observation group	45	21.98 ± 4.11	9.13 ± 1.21*	85.25 ± 5.09	64.34 ± 5.15*
t		1.186	5.123	0.033	4.406 <0.001
*P*		0.239	<0.001	0.974	

Compared with that before nursing, *P < 0.05.

### Inflammatory cytokine levels

After nursing interventions, the observation group had lower levels of CRP, IL-4, and PCT than the control group (*P* < 0.05) (Table 4).

**TABLE 4:** Comparison of inflammatory cytokine levels between the two groups ($\bar{x}$ ± s).

Group	Number of cases	CRP (mg/L)		IL-4 (ng/L)		PCT (ng/mL)	
		Pre-nursing	Post-nursing	Pre-nursing	Post-nursing	Pre-nursing	Post-nursing
Control group	45	40.89 ± 3.57	25.34 ± 3.70*	65.74 ± 3.65	50.21 ± 3.97*	19.25 ± 2.69	14.08 ± 2.49*
Observation group	45	40.79 ± 3.21	15.26 ± 3.11*	65.64 ± 3.56	35.34 ± 3.71*	19.26 ± 2.81	10.49 ± 2.52*
t		0.134	13.975	0.135	18.353	0.021	6.787 <0.001
*P*		0.984	<0.001	0.893	<0.001	0.983	

Compared with that before nursing, *P < 0.05.

### Nursing comfort

After nursing interventions, the observation group had higher GCQ scores across all dimensions and total scores than the control group (*P* < 0.05) (Table 5).

**TABLE 5:** Comparison of nursing comfort between the two groups ($\bar{x}$ ± s, points).

Group	Assessment time	Control group	Observation group	t	*P*
Physiology	Pre-nursing	26.29 ± 0.97	26.38 ± 0.89	0.454	0.651
	Post-nursing	34.18 ± 2.44*	42.89 ± 1.45*	20.572	<0.001
Psychology	Pre-nursing	29.62 ± 2.05	29.87 ± 1.95	0.58	0.563
	Post-nursing	38.31 ± 2.15*	44.89 ± 2.10*	14.67	<0.001
Sociocultural	Pre-nursing	15.38 ± 1.71	15.47 ± 1.84	0.237	0.813
	Post-nursing	20.53 ± 1.47*	22.44 ± 1.47*	6.164	<0.001
Environment	Pre-nursing	16.87 ± 2.11	16.73 ± 1.75	0.327	0.075
	Post-nursing	21.16 ± 1.99*	22.93 ± 1.14*	5.208	<0.001
Total score	Pre-nursing	88.16 ± 4.50	89.13 ± 3.14	1.195	0.235
	Post-nursing	114.18 ± 4.69*	133.16 ± 3.24*	22.343	<0.001

Compared with that before nursing, *P < 0.05.

### Patient satisfaction with nursing interventions

The observation group showed higher patient satisfaction with nursing interventions compared to the control group (*P* < 0.05) (Table 6).

**TABLE 6:** Comparison of satisfaction of the family members of patients between the two groups [*n* (%)].

Group	Number of cases	Very satisfied	Basically satisfied	Unsatisfied	Total satisfaction rate
Control group	45	8 (17.78)	25 (55.56)	12 (26.67)	33 (73.33)
Observation group	45	15 (33.33)	28 (62.22)	2 (4.44)	43 (95.56)
X^2^					32.352
*P*					<0.001

## Discussion

Ventilator-associated pneumonia is an avoidable complication caused by medical intervention, such as mechanical ventilation (17). Researchers currently focus global VAP prevention and treatment efforts on improving ventilated patients’ prognosis, particularly targeting mortality reduction and shorter hospital stays (18). Notably, nursing work leads the vast majority of VAP prevention measures (19), highlighting the critical role of nursing interventions in VAP prevention and control. This study reveals the impact of proactive risk-prevention nursing interventions on the recovery of ICU patients with VAP.

Our results showed that the observation group demonstrated notably shorter mechanical ventilation time, ICU stay, and body temperature recovery time than the control group, underscoring that proactive risk-prevention nursing interventions notably accelerates the recovery process of patients. These findings align with the research conclusion of Diana et al. (20), who pointed out that standardized nursing measures such as adopting a semi-recumbent position can effectively reduce the risk of VAP and shorten the duration of ventilatory support. The report by Li et al. (4) provides further evidence for our findings: health education and psychological support can effectively shorten hospital stays and optimize nursing outcomes. The risk-based personalized intervention system in this study, covering respiratory training for low-risk patients to nutritional support for high-risk patients, systematically enhanced the targeting and effectiveness of nursing, thereby promoting accelerated patient recovery.

After nursing interventions, both groups experienced a decrease in WBC, NC, CRP, IL-4, and PCT levels. However, the observation group demonstrated more pronounced reductions across all indicators. This suggests that proactive risk-prevention nursing interventions have a comparative advantage in regulating systemic inflammatory responses. This effect may stem from the comprehensive and personalized nursing system advocated by this model: for example, adjusting suctioning methods for medium-risk patients, providing phased nutritional support for high-risk patients, and systematically conducting psychological counseling. These measures create synergistic effects through multidisciplinary collaboration, collectively enhancing infection control and immune regulation, which aligns with the view proposed by Claudia et al. (21) that “targeted nursing interventions can enhance immune function and reduce infection rates.”

After nursing interventions, the observation group outperformed the control group in terms of scores on all dimensions of the GCQ and nursing satisfaction, indicating that this model has a significant effect in improving patients’ physical, psychological, and sociocultural comfort across multiple dimensions. This result can stem from the comprehensive and personalized nursing system: on one hand, standardized symptom management effectively alleviates patients’ physiological discomfort; on the other hand, psychological support measures integrated into daily nursing, such as channeling negative emotions through media, significantly enhance their psychological comfort levels, thereby forming a positive cycle between the body and mind. In addition, the improvement in patient satisfaction stems not only from the direct clinical outcome improvements they experienced (such as shortened ICU stays and symptom relief) but also from the emotional support provided through continuous communication during the nursing process. This holistic and humanized nursing model enhances patients’ trust and identification with the treatment process, thereby overall improving their nursing satisfaction. A previous study has shown that a nursing strategy that addresses patients’ physical, psychological, and cognitive needs can improve both care satisfaction, overall well-being and prognosis (22). Furthermore, by monitoring patient outcomes and implementing comprehensive, practical treatment protocols, this active strategy minimizes potential risks while optimizing rehabilitation outcomes (23, 24). Additional evidence indicates that proactive risk-prevention nursing interventions can significantly improve the quality of nursing care for children with bronchopneumonia, accelerate symptom relief, and result in high patient satisfaction (25).

In conclusion, this study demonstrates that proactive risk-prevention nursing interventions can reduce mechanical ventilation time, ICU stay duration, and inflammatory factor levels, while improving psychological comfort and nursing satisfaction in ICU patients diagnosed with VAP. However, the study still has certain limitations. As a single-center study, these findings require cautious interpretation regarding their generalizability. Future research should conduct multi-center, large-sample randomized controlled trials to further validate the universality and cost-effectiveness of this intervention model across different healthcare settings.

## Data Availability

The original contributions presented in this study are included in this article/supplementary material, further inquiries can be directed to the corresponding author.
